# Oral Environment of Esophageal Cancer Patients, the Incidence of Complications, and Long-Term Prognoses

**DOI:** 10.3390/curroncol33020086

**Published:** 2026-02-01

**Authors:** Yusuke Sato, Hiroki Nikawa, Akiyuki Wakita, Yushi Nagaki, Hiroshi Takano, Kazuhiro Imai

**Affiliations:** 1 Department of Esophageal Surgery, Akita University Hospital, Akita 010-8543, Japan; wakita@gipc.akita-u.ac.jp (A.W.); nagaki@med.akita-u.ac.jp (Y.N.); karo@doc.med.akita-u.ac.jp (K.I.); 2 Department of Thoracic Surgery, Akita University Graduate School of Medicine, Akita 010-8543, Japan; 3 Department of Dentistry and Oral Surgery, Hiroshima University Graduate School of Medicine, Hiroshima 734-8553, Japan; hirocky@hiroshima-u.ac.jp; 4 Department of Dentistry and Oral Surgery, Akita University Graduate School of Medicine, Akita 010-8543, Japan; htakano@med.akita-u.ac.jp

**Keywords:** esophageal cancer, esophageal squamous cell carcinoma, ESCC, periodontitis, oral condition, complication, prognosis, Toll-like receptor, TLR, *Porphyromonas gingivalis*

## Abstract

Recent studies have increasingly indicated that postoperative complications after esophagectomy are correlated with poor long-term prognoses, making it crucial to prevent such complications. Based on our studies, we believe that central to this issue is the finding that among esophageal cancer patients who experience postoperative complications and have poor long-term prognoses there is a high incidence of poor oral environments. Here we review the results of our basic and clinical research studies, as well as evidence from other institutions, on the oral environment of esophageal cancer patients and its association with the incidence of complications and long-term prognoses. We hope these findings, which suggest “improving the oral environment of esophageal cancer patients can reduce the incidence of postoperative complications and improve long-term prognoses”, will gain consensus and lead to safer esophageal cancer surgery.

## Simple Summary

We believe that the current consensus that “postoperative complications after esophagectomy are correlated with poor long-term prognoses” is related to the fact that many esophageal cancer patients have poor oral environments. We hope the new consensus “improving the oral environment of esophageal cancer patients can reduce the incidence of postoperative complications and improve long-term prognoses”, will gain consensus and lead to safer esophageal cancer surgery.

## 1. Introduction

Esophagectomy for esophageal cancer is a highly invasive procedure with a high risk of complications. The two most frequently occurring complications are anastomotic leakage and respiratory complications [1,2,3,4,5]. The recently reported incidences of anastomotic leakage ranges from 10% to 25%, while the incidences of respiratory complications are reportedly 15–40% [6]. Moreover, there is now a consensus that postoperative complications after esophagectomy are correlated with poor long-term prognoses, making it crucial to prevent such complications [7,8,9,10,11]. Based on our research, we believe a poor oral environment is a key condition in esophageal cancer patients that leads to higher rates of postoperative complications and poorer long-term prognoses. We have reported the results of a series of studies on the impact of the oral environment on the incidence of complications and long-term prognoses in esophageal cancer patients. It is our hope that the idea that “improving the oral environment in these patients will reduce the incidence of postoperative complications and improve long-term prognoses” will gain consensus and lead to safer esophageal cancer surgery. From that perspective, in this review we will summarize our basic and clinical research findings on the oral environment of esophageal cancer patients along with the accumulating evidence from other institutions. 

## 2. Periodontal Disease in Esophageal Cancer Patients

### 2.1. Esophageal Cancer Patients Have a High Incidence of Severe Periodontitis

**Figure 1:**
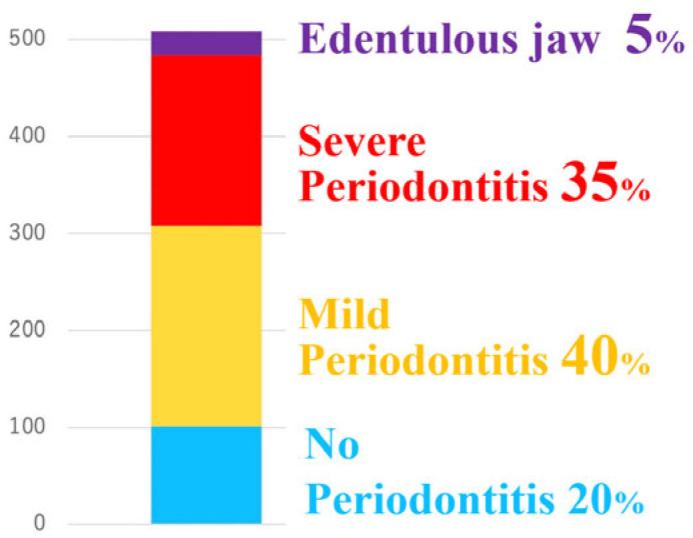
Results of preoperative oral assessment of 508 patients. Only 20% had no periodontitis, while 40% had mild periodontitis, 35% had severe periodontitis requiring tooth extraction, and 5% were already edentulous jaw.

A retrospective analysis of the 508 esophageal cancer patients who underwent preoperative oral function assessment by dentists between 2009 and 2021 in our hospital revealed that only 20% were assessed as having no periodontitis, while 40% had mild periodontitis, 35% had severe periodontitis requiring tooth extraction, and 5% already had edentulous jaw, having lost all their natural teeth. (Figure 1) In severe periodontitis patients, the average number of teeth extracted was 3.0, with a maximum of 22 teeth [12].

In recent years, numerous papers have been published on the relationship between periodontal disease and esophageal cancer, consistent with our clinical research findings. A recent large-scale, population-based case-control study in Korea demonstrated that edentulous jaw and periodontal disease were both significantly associated with increased odds of esophageal cancer [13]. Another large study in Sweden demonstrated that periodontal disease is significantly associated with increased odds of both esophageal squamous cell carcinoma (ESCC) and esophageal adenocarcinoma (EAC) [14]. Moreover, it is reported that individuals with lower economic status, smoking, heavy alcohol consumption, and irregular tooth brushing frequency were significantly associated with periodontal diseases and ESCC [15]. It is also reported that tooth loss and lack of regular oral hygiene are associated with a higher risk of ESCC [16]. Similarly, it is reported that periodontal disease and tooth loss are risk factors of EAC [17].

### 2.2. Preoperative Oral Care Reduces Postoperative Pneumonia in Esophageal Cancer Patients

Prior to 2009, our department performed esophagectomies for esophageal cancer without paying attention to the oral environment of these patients. During those years, the incidence of severe pneumonia requiring postoperative reintubation and ventilatory management was as high as 15.9% [18]. In this study, we also demonstrated that those cases developing severe pneumonia postoperatively had a significantly higher rate of anastomotic leakage. However, in 2009, after realizing that many esophageal cancer patients had a poor oral environment, we began performing preoperative oral function evaluations with dentists and tooth extractions, when necessary, before performing esophageal cancer surgery. Though there are also various other factors, this seems to dramatically reduce the incidence of severe pneumonia and there have been no such occurrences in the past five years [12].

Multiple studies from various institutions have demonstrated that preoperative oral care reduces postoperative pneumonia in esophageal cancer patients, consistent with our study [19,20,21,22,23]. On the other hand, no studies have directly demonstrated that preoperative oral care reduces anastomotic leakage after esophageal cancer surgery.

### 2.3. Severity of Periodontal Disease Is an Independent Risk Factor Affecting OS and DSS

**Figure 2:**
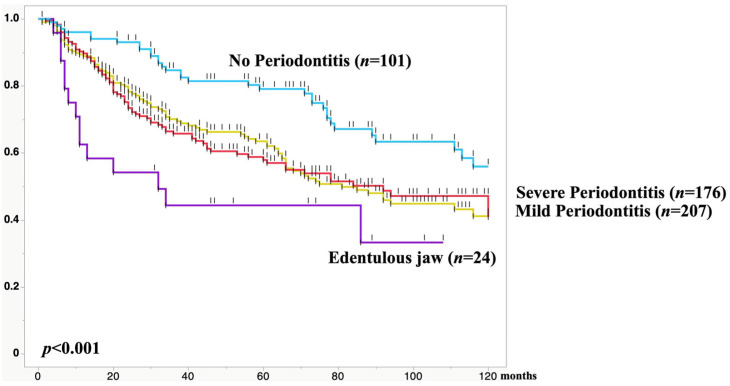
Comparison of 10-year OS in esophageal cancer patients segregated based on periodontitis severity. The best prognosis was observed in patients with no periodontitis, while the poorest prognosis was seen in edentulous jaw patients.

When we investigated whether the severity of periodontal disease affects long-term prognosis in these 508 esophageal cancer patients, we found that severity of periodontitis significantly affected both overall survival (OS) and disease-specific survival (DSS) [12]. (Figure 2) The best prognosis was observed in patients with no periodontitis, while the poorest prognosis was seen in edentulous jaw patients. Among these four groups—no periodontitis, mild periodontitis, severe periodontitis requiring tooth extraction, and edentulous jaw—the edentulous jaw group showed significantly higher age at surgery (70.7 vs. 65.1), significantly higher rate of current smoking habits (58.3% vs. 45.9%), and significantly lower rate of %VC over 80% (79.2% vs. 95.2%). Nevertheless, multivariate analysis showed that periodontal disease severity was an independent risk factor affecting OS and DSS. That was the first study to demonstrate that periodontal disease severity is an independent factor affecting long-term prognosis after esophageal cancer surgery.

Similarly, the number of lost teeth and the depth of periodontal pockets have been reported to be related to postoperative prognosis [24]. In this study, among 163 esophageal cancer patients who underwent surgery with perioperative oral care and examination, there was a significant difference in the 5-year OS rates between the groups: tooth loss < 13 and lower periodontal pocket index 74.8%, tooth loss < 13 and higher periodontal pocket index 62.8%, and tooth loss ≥ 13 50.5% (*p* = 0.0098).

Based on the results of these numerous clinical studies, it appears that a consensus is emerging that periodontal disease is a risk factor for esophageal cancer and is also related to postoperative pneumonia and its prognosis.

## 3. Periodontal Disease Bacteria and Esophageal Cancer

### 3.1. Periodontal Disease Bacteria Pyramid

**Figure 3:**
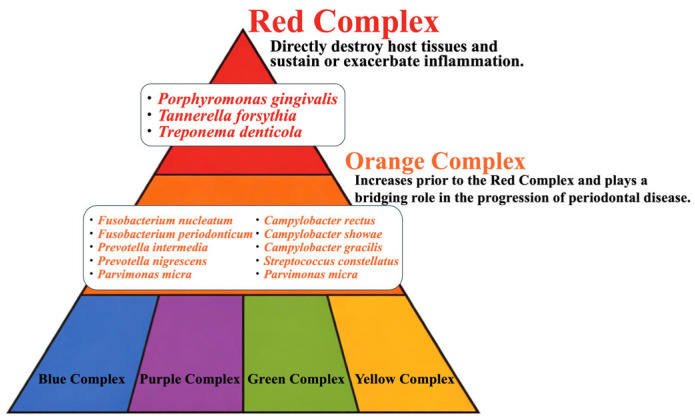
Periodontal disease bacteria pyramid. Representative “bad bacteria” are periodontal bacteria, which are classified as red complex, orange complex, yellow complex, etc., based on their association with periodontal disease.

Shifting perspective from macro to micro, there are about 700 kinds of bacteria in the human oral cavity, classified as “beneficial”, “opportunistic”, or “bad” bacteria. Representative bad bacteria are those that cause periodontal disease and are classified as red complex, orange complex, yellow complex, etc., based on their association with periodontal disease [25,26]. This is the so-called “periodontal disease bacteria pyramid” (Figure 3). The red complex consists of three species: *Porphyromonas gingivalis*, *Tannerella forsythia,* and *Treponema denticola*. These three species are all Gram-negative. It has been reported that the red complex causes inflammation and destroys the host’s tissues [27,28]. It has been reported that the orange complex increases before the red complex and plays a bridging role in periodontitis [25,29,30]. Likewise, 7 of 10 species of the orange complex are also Gram-negative. We therefore focused on the relationship between esophageal cancer and *Gram-negative bacteria* from a molecular oncological perspective.

### 3.2. Toll-like Receptor 4 Recognizes Lipopolysaccharide Released from Gram-Negative Bacteria

The most extensively studied receptors that recognize the structure of bacteria are the Toll-like receptors (TLRs) [31,32,33,34,35,36,37,38]. TLRs are important signaling molecules involved in innate immune responses and mediating antigen-specific adaptive immunity against multiple microbial pathogens. TLRs function as pattern recognition receptors able to recognize molecular structures known as pathogen-associated molecular patterns (PAMPs), which are delivered from a wide range of pathogens, including viruses, bacteria, fungi, and parasites [31,32]. Because it is well known that lipopolysaccharide (LPS) released from the *Gram-negative bacterial* cell wall is recognized by TLR4, we investigated TLR4 expression in ESCC tissue and its relationship to prognosis.

### 3.3. High TLR4 Expression in ESCC Predicts a Poor Prognosis

**Figure 4:**
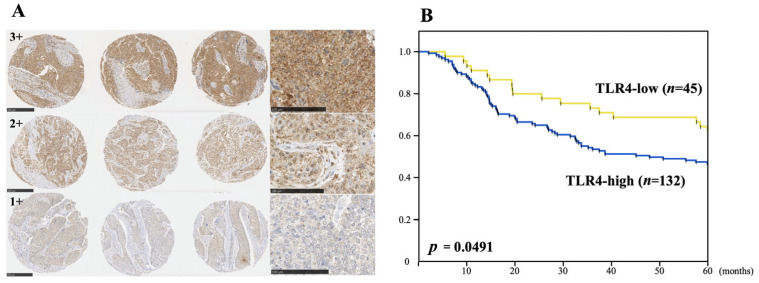
High TLR4 expression in ESCC predicts a poor prognosis. (**A**): Immunohistochemical staining of TLR4 in an ESCC tissue microarray (TMA). The representative photomicrographs show triplicate specimens scored 3+, 2+ or 1+. There were no completely negative specimens. Whole triplicate cores from an ESCC TMA are shown at 100× magnification, along with high-magnification (400×) images on the right. (**B**): Kaplan–Meier curves illustrating the association between the TLR4 expression status (high or low) and 5-year OS in ESCC patients after esophagectomy. “Adapted with permission from Ref. [39]. 2023, Springer Nature”.

We first assessed whether the expression status of TLR4 in patients with ESCC correlated with their prognosis after esophagectomy [39]. Clinical ESCC samples from all 177 patients tested showed expression of TLR4. Moreover, high TLR4 expression (3+ and 2+) correlated with poorer 5-year OS after esophagectomy than lower TLR4 expression (1+). (Figure 4) Patients showing high TLR4 expression tended to have a poorer prognosis whether treated with surgery alone or with surgery and adjuvant chemotherapy. Univariate and multivariate analyses showed TLR4 expression status to be an independent prognostic factor affecting 5-year OS.

### 3.4. “Beneficial Bacteria” Are Gram-Positive Bacteria

While most periodontal disease bacteria are Gram-negative, most beneficial bacteria residing in the human oral cavity are Gram-positive [40,41]. The best studied beneficial bacterium is *Streptococcus mitis* [42,43]. This is the species that infants receive from their mothers through breast milk. Infants’ abundant saliva contains a potent antimicrobial substance, defensin, which prevents most bacteria from colonizing their oral cavity. However, *S. mitis* is resistant to defensin and is one of the few bacteria capable of colonizing the infant’s oral cavity. Furthermore, as baby teeth emerge, *S. mitis* coats them, preventing other bacteria from adhering. They are therefore referred to as “early colonizing bacteria”. Notably, *S. mitis* is Gram-positive, as are other beneficial bacteria, including *Streptococcus oralis* and *Streptococcus salivarius*. Similarly, bacteria that play central roles in food fermentation—such as certain lactate-producing bacteria, certain butyrate-producing bacteria, and certain Bacillus subtilis bacteria, such as *natto bacteria*—are all Gram-positive [44].

### 3.5. High TLR6 Expression in ESCC Predicts a Better Prognosis

**Figure 5:**
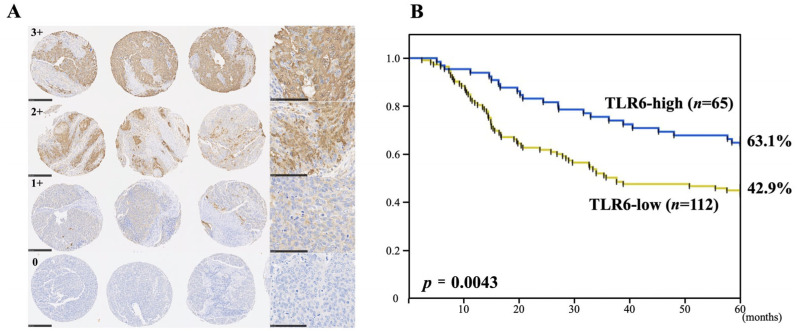
High TLR6 expression in ESCC predicts a better prognosis. (**A**): Immunohistochemical staining of TLR6 in selected ESCC cores. Shown are photomicrographs of representative triplicate specimens scored 3+, 2+, 1+, or 0. The triplicate cores are shown at 100× magnification, along with high-magnification (400×) images on the right. (**B**): Kaplan–Meier curves illustrating the association between the TLR6 expression status (high or low) and 5-year OS in ESCC patients after esophagectomy. “Adapted with permission from Ref. [45]. 2020, MDPI”.

While TLR4 recognizes LPS, TLR2/6 heterodimers reportedly recognize peptidoglycan (PGN) released from Gram-positive bacteria [32]. We therefore assessed the expression status of TLR2 and TLR6 in patients with ESCC to determine whether their expression status correlated with patient prognosis after esophagectomy. Although TLR2 expression showed no correlation with postoperative prognosis after esophageal cancer surgery, high TLR6 expression (3+ and 2+) correlated with significantly more favorable 5-year overall and disease-specific survival after esophagectomy than lower TLR6 expression (1+ and 0) [45]. (Figure 5) Moreover, univariate and multivariate analyses showed TLR6 expression status to be an independent prognostic factor affecting 5-year OS.

## 4. How to Create Beneficial Bacteria Predominate Environment in the Mouth

### 4.1. How Can Esophageal Cancer Patients Create an Environment in Their Mouth Where Beneficial Bacteria Predominate?

Given the findings summarized above, it appeared to us that the balance of oral bacteria in esophageal cancer patients likely influences postoperative prognosis. This suggested that if we could create an environment dominated by beneficial bacteria in the oral cavity of esophageal cancer patients, it may not only reduce postoperative complications but also decrease postoperative recurrence and improve prognosis. And just as we were considering how to efficiently create an environment in the mouth where beneficial bacteria predominate, we noticed an oral care product containing *Lacticaseibacillus rhamnosus L8020*.

### 4.2. Lacticaseibacillus rhamnosus L8020

*Lacticaseibacillus rhamnosus L8020* is a strain of lactic acid bacteria discovered by Professor Hiroki Nikawa of the Department of Dentistry, Hiroshima University. To address the simple question, “Why is it that some people never get cavities or periodontal disease even if they don’t brush their teeth?”, he isolated 42 types of lactic acid bacteria present in Japanese human oral cavities. From among them, he selected *L. rhamnosus L8020*, which exhibited the greatest ability to eliminate both dental caries bacteria and periodontal disease bacteria constituting the red complex [46]. They proved in this in vitro study that yogurt containing *L. rhamnosus L8020* significantly reduced Streptococcus mutans and four periodontal pathogens examined—*Porphyromonas gingivalis*, *Prevotella intermedia*, *Tannerella forsythia*, and *Fusobacterium* spp.—but the phenomenon were not observed with the placebo yogurt. They also proved that *L. rhamnosus L8020* significantly reduced the abundance of periodontal pathogens in individuals with intellectual disability in the randomized clinical trial.

**Figure 6:**
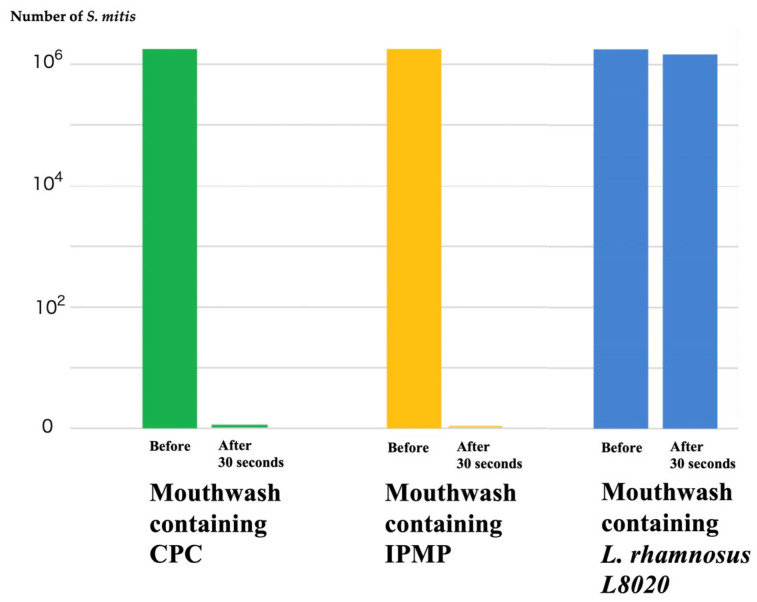
Mouthwash containing CPC or IPMP eliminates S. mitis. Mouthwashes containing strong antibacterial agents such as CPC and IPMP are eliminating not only periodontal bacteria but also beneficial bacteria in the oral cavity. (Testing Agency: Japan Food Research Laboratories).

Though following results have not been reported in English-language papers, he also discovered that two specific peptides released from *L. rhamnosus L8020* had the ability to eliminate major dental caries bacteria, periodontal disease bacteria, and *Candida albicans*. Toothpaste and mouthwash containing *L. rhamnosus L8020* are already commercially available in Japan and have been shown to significantly reduce periodontal disease bacteria and *Candida albicans* without affecting beneficial bacteria in the oral flora, including *S. mitis* and *S. oralis*. On the other hand, surprisingly, many commercially available mouthwashes, including cetylpyridinium chloride (CPC) or isopropyl methylphenol (IPMP), have been proven to eliminate *S. mitis* within 30 s. (Figure 6) Based on these results, mouthwashes containing strong antibacterial agents such as CPC and IPMP are eliminating not only periodontal bacteria but also beneficial bacteria in the oral cavity.

### 4.3. Clinical Intervention with Toothpaste and Mouthwash Containing L. rhamnosus L8020

Based on our earlier findings and the discovery of oral care products containing *L. rhamnosus L8020*, we initiated a clinical trial: “Improvement of oral environment with toothpaste and mouthwash containing *L. rhamnosus L8020* and the incident rate of pneumonia after esophagectomy for esophageal cancer patients” (LacPEC study, jRCTs021230010) in June 2023. Although we are still accumulating cases, interim analysis elucidated that the incidences of pneumonia (Clavien-Dindo grade 2 and more) and atelectasis (Clavien-Dindo grade 3a and more) are significantly lower in the group whose oral care includes *L. rhamnosus L8020* than in the group without it. Moreover, the incidence of anastomotic leakage (Clavien-Dindo grade 1 and more) is also significantly lower in the *L. rhamnosus L8020* group (*p* = 0.048). These results suggest a mechanism whereby periodontal bacteria infect the mucosal layer and muscular layer immediately after anastomosis, before the barrier is fully established, causing these layers to become vulnerable and leading to anastomotic failure. Thus far, the results of this clinical trial suggest that creating an oral environment where “beneficial bacteria” predominate before esophagectomy is a valid means of reducing postoperative complications.

## 5. Basic Research About Bacteria and ESCC Cell Proliferation

### 5.1. Periodontal Bacteria Promote the Proliferation of Esophageal Cancer Cells

As mentioned earlier, the available evidence indicates that periodontitis is a risk factor of esophageal cancer [13,14,15,16,17]. The most extensively studied periodontal bacteria is *Porphyromonas gingivalis*, classified within the red complex. Studies have shown that *P. gingivalis* induces malignant transformation of normal esophageal epithelium [47]. It is also associated with lymph node metastasis and reduced survival time in ESCC [48], ESCC progression that can be effectively suppressed by promoting its removal [49], and reduced sensitivity to chemotherapy in ESCC [50]. We further showed that the level of *P. gingivalis* infection correlates with TLR4 expression status of ESCC and predicts a poorer prognosis in ESCC patients after esophagectomy [51]. Univariate and multivariate analyses showed that the *P. gingivalis* status is an independent prognostic factor affecting 5-year OS and DSS. Moreover, the combined *P. gingivalis* and TLR4 statuses are also an independent prognostic factor affecting 5-year OS and DSS. Based on these recent findings, preventing *P. gingivalis* infection may be an effective strategy for improving the long-term outcomes of patients with ESCC.

The next most well studied periodontal bacterium is *Fusobacterium nucleatum*, classified within the orange complex. Previous studies revealed that *F. nucleatum* infection predicts a poor prognosis in ESCC patients [52] and is associated with reduced sensitivity to chemotherapy in ESCC [53]. Another periodontal bacterium, *Tannerella forsythia*, classified within the red complex, is reported to be a risk factor for EAC with a high odds ratio [54]. All of these studies demonstrate that periodontal bacteria exert promotive effects on esophageal cancer cell proliferation, while no studies have demonstrated an inhibitory effect on esophageal cancer cell proliferation.

### 5.2. LPS Constituting the Cell Wall of Gram-Negative Bacteria Promotes ESCC Cell Proliferation

Given the results of the studies summarized above and others, we hypothesized that LPS constituting the cell wall of Gram-negative bacteria promotes the proliferation of ESCC. Consistent with that idea, our recent study revealed that LPS significantly upregulates cell proliferation and tumor progression through an LPS-TLR4-CCL2 cascade and influences prognosis after esophagectomy for ESCC [55]. Moreover, in a mouse xenograft model, subcutaneous LPS administration significantly increased ESCC tumor volume. This suggests reducing periodontal bacteria in the oral environment has the potential to improve the prognosis of ESCC patients after esophagectomy. Importantly, it also suggests that reducing periodontal bacteria in oral environment of healthy individuals could potentially reduce their risk for ESCC.

### 5.3. PGN Constituting the Cell Wall of Gram-Positive Bacteria Inhibits ESCC Cell Proliferation

On the other hand, we also hypothesized that PGN constituting the cell wall of Gram-positive bacteria inhibits ESCC cell proliferation, and our recent study confirmed that PGN significantly suppresses ESCC cell proliferation [56]. We also revealed that PGN upregulates CXCL10 production via TLR6 signaling and promotes apoptosis of ESCC cells. Moreover, in a mouse xenograft model, subcutaneous PGN administration tends to decrease ESCC tumor volume.

### 5.4. The Ability of Specific Peptides Produced by L. rhamnosus L8020

Professor Nikawa’s studies show that two specific peptides produced by *L. rhamnosus L8020* exert antibacterial effects against dental caries and periodontal disease bacteria. Moreover, these peptides are able to inactivate LPS released from *P. gingivalis* and suppress the secretion of inflammatory cytokines from gingival fibroblasts in a concentration-dependent manner. In addition, our recent experiments newly revealed that these peptides significantly inhibit proliferation of 14 ESCC cell lines and 3 EAC cell lines, as well as lung adenocarcinoma, gastric cancer, and pancreatic cancer cell lines (cannot be disclosed due to patent application matters). These peptides seem to hold potential as growth inhibitors for multiple cancers.

## 6. Periodontal Disease Adversely Affects Numerous Systemic Diseases Other than Esophageal Cancer

It has been revealed that periodontal disease is associated not only with esophageal cancer but also with numerous systemic diseases [57,58,59]. Vascular diseases including ischemic heart disease, cerebral infarction/stroke, and atherosclerosis, type 2 diabetes and diabetic complications, respiratory diseases including aspiration pneumonia and COPD, pregnancy and perinatal complications including preterm birth and preeclampsia, numerous cancers including esophageal cancer, chronic kidney disease, autoimmune diseases including rheumatoid arthritis and systemic lupus erythematosus, metabolic syndrome including obesity and osteoporosis, and neurological and psychiatric disorders including Alzheimer’s disease, dementia, and Parkinson’s disease, are representative examples. The mechanisms by which periodontal bacteria adversely affect these diseases are also becoming understood, and *L. rhamnosus L8020*, which can eliminate periodontal bacteria, are highly likely to alleviate these diseases or reduce the risk of developing them.

## 7. Discussion

This review has summarized, based on numerous previous studies, that periodontal disease is a risk factor for esophageal cancer and further influences the incidence of postoperative complications and long-term prognosis following esophageal cancer surgery. None of the previously reported papers suggesting that postoperative complications of esophageal cancer affect long-term prognosis have mentioned the oral environment of such patients. We hope this review contributes to the spread of the consensus that “improving the oral environment of esophageal cancer patients can reduce the incidence of postoperative complications and improve long-term prognoses”.

**Figure 7:**
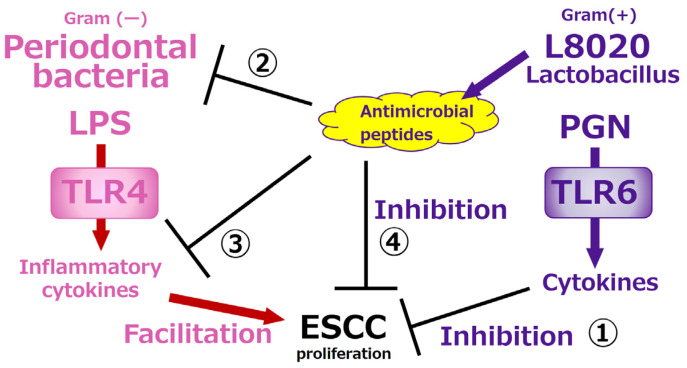
The mechanisms that have been clarified so far. There are at least four mechanisms by which *L. rhamnosus L8020* exert inhibitory effects on ESCC cell proliferation.

The results of our basic studies and those performed by Professor Nikawa suggest there are at least four mechanisms by which *L. rhamnosus L8020* exert inhibitory effects on ESCC cell lines (Figure 7). First, *L. rhamnosus L8020* are Gram-positive, and the PGN that constitutes their cell wall suppresses the proliferative capacity of ESCC [56]. Second, periodontal disease bacteria are Gram-negative, and the LPS that constitutes their cell wall enhances the proliferative capacity of ESCC cells [55]. However, specific peptides produced by *L. rhamnosus L8020* eliminate the periodontal disease bacteria [46]. Third, these specific peptides produced by *L. rhamnosus L8020* also have ability to inactivate LPS and suppress secretion of inflammatory cytokines from gingival fibroblasts. Fourth, these peptides are able to inhibit proliferation of ESCC cell lines directly. The elucidation of these mechanisms suggests that using oral care products containing *L. rhamnosus L8020* may not only reduce postoperative complications in esophageal cancer patients, but it may also decrease postoperative recurrence, improve prognosis, and potentially lower the risk of esophageal cancer in healthy individuals.

Elucidation of this mechanism suggests that oral care containing *L. rhamnosus L8020* may not only enhance the efficacy of surgical treatment for esophageal cancer but also potentially boost the effects of chemotherapy, radiation therapy, and immune checkpoint inhibitors. Recent advances in these non-surgical treatments have been remarkable, and the long-term prognosis for esophageal cancer patients overall has improved. Future clinical trials should also clarify whether containing *L. rhamnosus L8020* enhances the efficacy of these treatments.

Moreover, periodontal disease has been reported to be associated not only with esophageal cancer but also with numerous systemic diseases. Determining whether oral care containing *L. rhamnosus L8020* actually reduces the risk of esophageal cancer and numerous systemic diseases will require performing large-scale prospective cohort studies in the future.

## 8. Conclusions

Based on observations that esophageal cancer patients often have poor oral environments, our clinical and basic research revealed that establishing an oral environment dominated by beneficial bacteria likely offers multiple advantages. These include not only preventing postoperative complications but also suppressing postoperative recurrence, improving prognosis, and reducing the risk of esophageal cancer. Our interim analysis of clinical trial demonstrates that creating a beneficial bacterial environment in the oral cavity of esophageal cancer patients significantly reduces the risk of postoperative pneumonia and anastomotic leakage. However, whether it also improves the prognosis after esophageal cancer surgery or reduces the risk of esophageal cancer requires demonstration in large-scale prospective cohort studies.

## 9. Future Directions

Our interim analysis of clinical trial thus far suggests that using oral care products containing *L. rhamnosus L8020* before esophagectomy is a valid means of reducing the likelihood of postoperative complications. We anticipate that whether *L. rhamnosus L8020* actually reduce postoperative recurrence, improve prognoses, and lower the risk of esophageal cancer and numerous systemic diseases will be determined in future large-scale prospective cohort studies. We hope that the idea that “improving the oral environment of esophageal cancer patients reduces the incidence of postoperative complications and improves their long-term prognosis” will gain consensus and lead to safer esophageal cancer surgery.

## Data Availability

The original contributions presented in this study are included in the article. Further inquiries can be directed to the corresponding author.

## Abbreviations

The following abbreviations are used in this manuscript:

ESCC	esophageal squamous cell carcinoma
EAC	esophageal adenocarcinoma
LPS	lipopolysaccharides
PGN	peptidoglycan
CCL2	C-C motif chemokine ligand 2
CXCL10	C-X-C motif chemokine ligand 10
TLR4	Toll-like receptor 4
TLR6	Toll-like receptor 6
